# 

**Published:** 1829-07-01

**Authors:** 


### MEDICO-CHIRURGICAL

### REVIEW,

AND

### JOURNAL

OF

### PRACTICAL MEDICINE.

*(NEW SERIES.)*

VOLUME ELEVEN,

[1st of APRIL, to 30th of SEPTEMBER,]

1829.

VOL. XV. of ANALYTICAL SERIES.

EDITED BY JAMES JOHNSON, M.D.

&c. &c. &c.

Surgeon Genl's Office
LIBRARY
57459
Washington, D.C.

LONDON:

PUBLISHED BY S. HIGHLEY, 174, FLEET STREET,

AND WEBB STREET, ST. THOMAS'S HOSPITAL,

And all other Booksellers.

W1
ME 15T

PRINTED BY G. HAYDEN,

Little College Street, Westminster.

### BIBLIOGRAPHICAL RECORD.

1. The Anatomy of Drunkenness. By Robert Macnish, Member of the Faculty of Physicians and Surgeons of Glasgow. Third Edition. 12mo, pp. 216, Glasgow, 1829.

2. Cours de Physiologie Générale et Comparée Professé à la Faculté des Sciences de Paris. Par M. Ducrotay de Blainville, &c. &c. Livraison, 8vo, Paris, 1829.

3. No. 3 of the Annals of Chemical Philosophy, &c. By Wm. Maugham, Surgeon, Lecturer on Chemistry, Materia Medica, &c. 8vo, pp. 240, price 2s. 6d. April, 1829.

4. The Study of Medicine. By John Mason Good, M.D. F.R.S. &c. Containing all the author's final corrections and improvements. Third Edition, with much additional modern information on Physiology, Practice, Pathology, and the Nature of Diseases in general. By Samuel Cooper, Surgeon to the King's Bench and Fleet Prisons; Surgeon to the

Forces, &c. &c. In five volumes, 8vo, 1829. Uuderwoods.

5. Pathological Observations, Part 2, on Continued Fever, Ague, Tic Douloureux, Measles, Small Pox, and Dropsy, &c. By WILLIAM STOKER, M.D. &c. 8vo, pp. 267. 1829.

6. Harveian Oration for MDCCCXXIX; being a Tribute of Respect for the Memory of the late Andrew Duncan, Sen. M.D. By RICHARD HUIE, M.D. &c. &c. 8vo, stitched, pp. 28, Edinburgh, 1829.

7 An Essay on the Symptoms of Pregnancy, from the Earliest Stage to the Period of Quickening, &c. to which was awarded Dr. Hopkins's Prize Gold Medal for 1828-9. By JOHN MORLEY. 8vo, stitched, pp. 48. London, 1829.

☞ *A very creditable Essay to the author, and one which well deserved the honourable mark of distinction conferred on it by that zealous teacher, Dr. Hopkins.*

8. The Evidences against the System of Phrenology, being the Substance of a Paper read at an Extraordinary Meeting of the Royal Medical Society of Edinburgh. By THOMAS STONE, Esq. 8vo, pp. 75. Edinburgh, 1828.

☞ *Without entering into the controversy, we will venture to say that Mr. Stone has evinced great research and literary talent of a very high order in the composition of this work. We hope to see the same research and the same talents employed on more strictly professional subjects.*

9. The Influence of Climate in the Prevention and Cure of Chronic Diseases, more particularly of the Chest and Digestive Organs, &c. with an Appendix containing a Series of Tables on Climate. By JAMES CLARK, M.D. &c. 8vo, pp. 328. London, 1829.

10. A Treatise on Syphilis; in which the History, Symptoms, and Method of Treating every Form of that Disease, are fully considered. By JOHN BACOT, Surgeon to the St. George's and St. James's Dispensary, and lately Surgeon to the Grenadier Regiment of Foot Guards. 8vo, pp. 280, London, 1829.

11. Travels in Turkey, Egypt, Nubia, and Palestine, in 1824-5-6 and 7. By R. R. MADDEN, Member of the Royal College of Surgeons. In two volumes Octavo. June, 1829.

12. Management and Diseases of Infants under the Influence of the Climate of India, &c. By FRED. CORBYN, Esq. 8vo. Calcutta, 1828.

13. Anti-Phrenology, &c. By JOHN WAYTE, M.D. 8vo, pp. 97. Lynn Regis, June, 1829.

14. Scriptores Ophthalmologici Minores. Vols. 1 and 2. Edidit JUSTUS RADIUS, Philos. Med. et Chir. Doct. &c. Cum Tabulis Æneis. Lipsiæ, 1826-1828.

☞ *Germany is famous for learned physicians, and Dr. Julius is justly esteemed one of her present luminaries. The two small volumes above-mentioned are written in classical Latin, and contain a great body of important information on Ophthalmological subjects, together with numerous references to the best writers on the Diseases of the Visual Apparatus, and the Operations necessary for their Cure.*

15. Observations on Derangements of the Digestive Organs, and on their Connexion with Local Complaints. By WILLIAM LAW, Esq. 2nd Ed. enlarged. 1829.

16. Elements of Medical Statistics; containing the Substance of the Gulstonian Lectures delivered at the College of Physicians; with numerous additions, &c.. &c. &c. By F. BISSET HAWKINS, M.D. of Exeter College, Oxford, &c. 8vo, pp. 234. 1829.

17. On the Varieties of Deafness, and Diseases of the Ear, with proposed Methods of relieving them. By WILLIAM WRIGHT, Esq. Surgeon Aurist to her late Majesty Queen Charlotte, &c. &c. 8vo, pp. 295, London, 1829.

18. An Account of the Morbid Appearances exhibited on Dissection in Disorders of the Trachea, Lungs and Heart, with Pathological Observations, to which a Comparison of the Symptoms with the Morbid Changes has given rise. By THOMAS MILLS, M.D. Honorary Fellow of the King and Queen's College of Physicians. 8vo. pp. 302. Dublin and London, 1829.

19. The Anatomy, Physiology, and Diseases of the Teeth. By THOMAS BELL, F.R.S. F.G.S. &c. Lecturer at Guy's Hospital, and Surgeon-Dentist to that Institution. 8vo, pp. 330, Eleven Plates. Highley, London, April, 1829.

